# The Femoral Neck-Bite Sign: A Radiographic Indicator of Catastrophic Sandwich Liner Failure in Total Hip Arthroplasty

**DOI:** 10.1016/j.artd.2025.101740

**Published:** 2025-06-23

**Authors:** Hendrik Pott, Ricarda Stauss, Peter Savov, Max Ettinger, Ralf Dieckmann

**Affiliations:** a Division of Orthopaedics at Campus Pius-Hospital, Carl von Ossietzky Universität Oldenburg, School of Medicine and Health Sciences, Oldenburg, Germany; b Department of Orthopedic Surgery, Krankenhaus der Barmherzigen Brüder Trier, Trier, Germany

**Keywords:** Sandwich type liner, Ceramic liner fracture, Femoral Neck-Bite Sign, Total hip arthroplasty revision, Case report

## Abstract

In this case report, we present the failure of a sandwich liner resulting in extensive notching of the femoral neck as well as metallosis and osteolysis in a 74-year-old woman. All implants were removed due to large bone defects and a cage was combined with a polyethylene cup, ceramic head, and modular cementless stem. Clinical and radiological follow-ups were conducted at 6 weeks and 6 months postoperatively, showing postoperative patient-reported outcome measures that were marginally inferior to the preoperative status. We discussed that a timely revision surgery was nonetheless warranted in order to prevent further bone loss and systemic toxic metallosis. We introduce the Femoral Neck-Bite Sign, a radiographic finding that may indicate ceramic liner fracture as in this case or implant impingement resulting in femoral notching.

## Introduction

Not only the number of young patients receiving a total hip arthroplasty, but also the number of older patients with a high activity index and functional demand, rises [[1], [2], [3], [4], [5]].

The use of a ceramic-on-ceramic bearing thereby becomes more and more attractive, facilitating low surface roughness, major scratch resistance, and a high wettability, thus suggesting an increased durability compared to other types of bearing [[6], [7], [8]]. However, registry data show that there is a trend from a ceramic-on-ceramic bearing surface toward an increased use of ceramic-on-cross-linked polyethylene articulation [3,7,9].

Main points of criticism of the ceramic-on-ceramic bearing are the risk for fracture of the ceramic head or liner and noise development, such as squeaking [[10], [11], [12]].

Starting in 1994, a sandwich type liner was introduced that combined a ceramic and polyethylene liner in a titanium cup. This compromise promised the low friction of the ceramic-on-ceramic bearing combined with a less rigid fit in the cup [13].

In the following years, multiple studies reported early failure and fracture of the sandwich type liner with only few studies showing favorable results [13,14]. The use of this type of liner was largely discontinued, but in patients who have received this implant we can still assume a high rate of liner failure in the present [[15], [16], [17]]. Shin et al. performed a review of 134 patients (143 hips) of which 6.2% had a liner fracture at a mean follow-up time of 126 months [15]. Sandwich and ceramic liners produce ceramic shards if fractured, both ultimately leading to the same surgical challenges that are metallosis and osteolysis [18]. In this case report, we present a patient featuring several remarkable findings in the context of a sandwich type liner fracture.

## Case history

### Patient information

**Figure 1:**
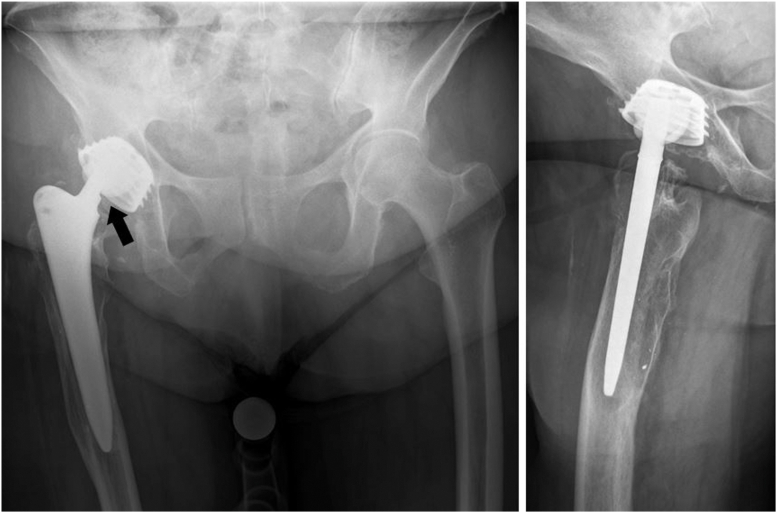
Preoperative plain radiographs, anteroposterior, and right axial pelvis radiographs taken 4 years prior to the patient presented at our institution. Findings indicative of ceramic liner fracture include the head’s asymmetric position in relation to the cup and the Femoral Neck-Bite Sign signifying damage to the stem due to abrasions (black arrow). Large osteolytic areas are present already.

A 74-year-old Caucasian woman presented to our outpatient clinic complaining about pain in her right knee due to arthritic joint disease. A total hip arthroplasty had been performed on her right hip in 2003 (Fa. Stratec Medical, Screw-In Cup 50mm, Ceraminlay, Ceramic Head 28 mm) 20 years prior to her presentation at our institution. A scheduled total knee arthroplasty in another hospital had not been realized due to considerations regarding the COVID pandemic 4 years earlier. Standardized radiographs of knee and hip had been obtained (Fig. 1), an observed wear of the acetabular liner had not been addressed at the time according to the patient, since she did not experience any symptoms regarding her hip. At the time of her presentation at our institution, she still did not report any pain in her hip.

### Clinical findings

On clinical examination, we saw a patient who entered the examination room with a slightly antalgic gait but without walking aids. She was classified as ASA 1 on the ASA risk classification by the American Society of Anesthesiologists. Skin and soft tissues were intact; a well-healed scar on her right hip was evident after a prior lateral incision. The range of motion of the right hip was 100 degrees of flexion, full extension, 30 degrees of external and 20 degrees of internal rotation. There was no pain on internal rotation and no tenderness upon palpation of the greater trochanteric tubercle or groin. No instability or pain regarding the right hip could be provoked during the course of our clinical examination.

The mobility of the right knee ranged from full extension to 120 degrees of flexion, the patient reported pain on hyperflexion as well as on palpation of the medial and lateral femorotibial compartment. No palpable joint effusion was evident. There was no ligamentous instability and no pain upon palpation of the patellar. We found a reduced quadriceps muscle strength equivalent to a grade 3 on the Medical Research Council scale. This was concurrent with a previously described femoral nerve lesion that occurred during the total hip arthroplasty 20 years ago according to the patient. There was no decrease in sensation or further decrease of power concerning other muscle groups. The left hip and knee were both unremarkable on clinical examination.

### Diagnostic assessment

On initial plain radiographs of the pelvis as well as a right-sided axial view, an asymmetrical position of the femoral head in relation to the liner had been observed (Fig. 1).

Osteolytic areas were present in Charnley regions 1 and 7 to 14 as well as Greens regions 2 and 3 [19,20].

**Figure 2:**
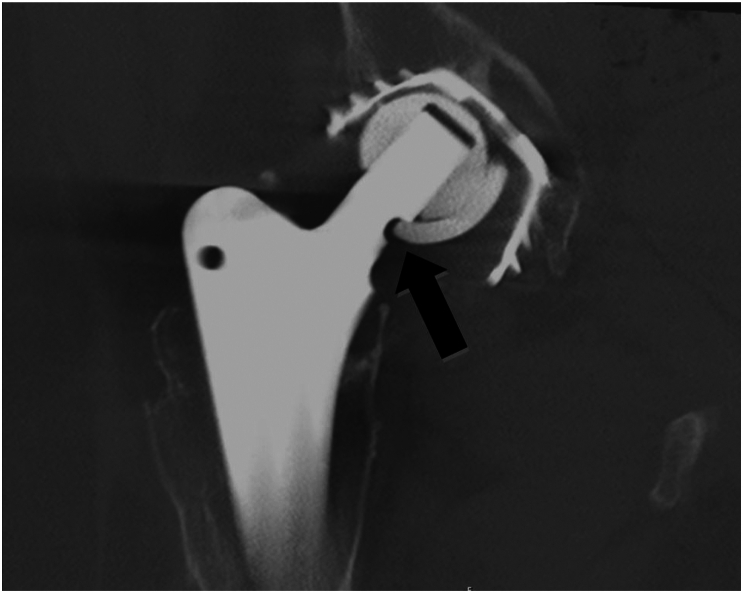
Computed tomography of the right hip, CT scan showing the Femoral Neck-Bite Sign (black arrow) due to a ceramic liner fracture, note the large osteolytic areas extending from the cup into the pelvis and surrounding the stem. Also of interest is a thinning of the acetabular cup in its superior aspect presumably caused by abrasive wear. The cup inclination as measured in the CT scan is 47 degrees and cup anteversion is 22 degrees. CT, computed tomography.

Furthermore, a notching of the femoral stem’s neck portion was apparent. The computed tomography scan performed at our institution confirmed a fracture of the ceramic liner and notching due to fragments which pressed into the femoral neck (Fig. 2).

This radiological finding, both present in plain X-rays and the computed tomography scan was reminiscent of a “bite” taken out of the stem’s neck portion. Thus, we named it the Femoral Neck-Bite Sign for its illustrative and educational value.

Synovial fluid aspiration of the right hip revealed a synovial white body count of 2.270/μl with a polymorphonuclear count of 55 percent. Microbiological cultures remained negative.

### Therapeutic intervention

We discussed the clinical and radiological findings with the patient and explained, that a fracture of the ceramic insert had occurred, not only damaging the femoral stem but also causing an at least partial loosening of both cup and stem, thus putting her at risk of developing extensive osteolysis. In an informed consent setting, we further discussed different strategies and ultimately decided in agreement with the patient that a timely revision of the right hip should be performed prior to any knee surgery in order to avoid further bone loss.

### Surgical procedure

The surgery was performed by the senior author (R.D.) at his institution. The patient was positioned in a supine position. Skin disinfection and surgical draping were performed. A skin incision was made while simultaneously excising the visible scar on the right hip, appreciating the previously used lateral approach.

On opening the fascia and underlying muscle, severe metallosis was observed. The gluteus medius showed signs of muscle atrophy as well as muscle degeneration. An extensive release was necessary to luxate the implant. After removal of the ceramic head, the abrasion and notching of the femoral neck due to impinging fragments of the ceramic liner was visible.

The bone surrounding the femoral stem offered large osteolytic areas due to metallosis in the proximal aspect of the femur and fractured on its dorsal side upon chiseling. The stem was eventually removed, thereby allowing inspection of the acetabular cup. The ceramic liner was severely fractured, and all visible fragments subsequently removed. Multiple samples were obtained for histopathological as well as microbiological analysis.

After removal of the cup, large osteolytic defects were observed on the central aspect of the acetabulum as well as the anterior acetabular rim, causing a segmental anterior pelvic defect.

For total hip arthroplasty revision, a Burch-Schneider cage construct was used. Defects were filled with absorbable calcium sulfate biocomposite (Stimulan, Biocomposites Ltd., Keele, UK). A polyethylene cup was then cemented into the cage.

Two harnessing cerclage cables were used on the proximal femur, followed by reaming, implantation of a modular cementless stem under fluoroscopic control, and addition of the modular components as well as a ceramic head. An additional prophylactic cerclage cable was added. A drain was introduced, the layers of tissue were subsequently closed and intraarticular tranexamic acid was applied.

### Postoperative care regimen

**Figure 3:**
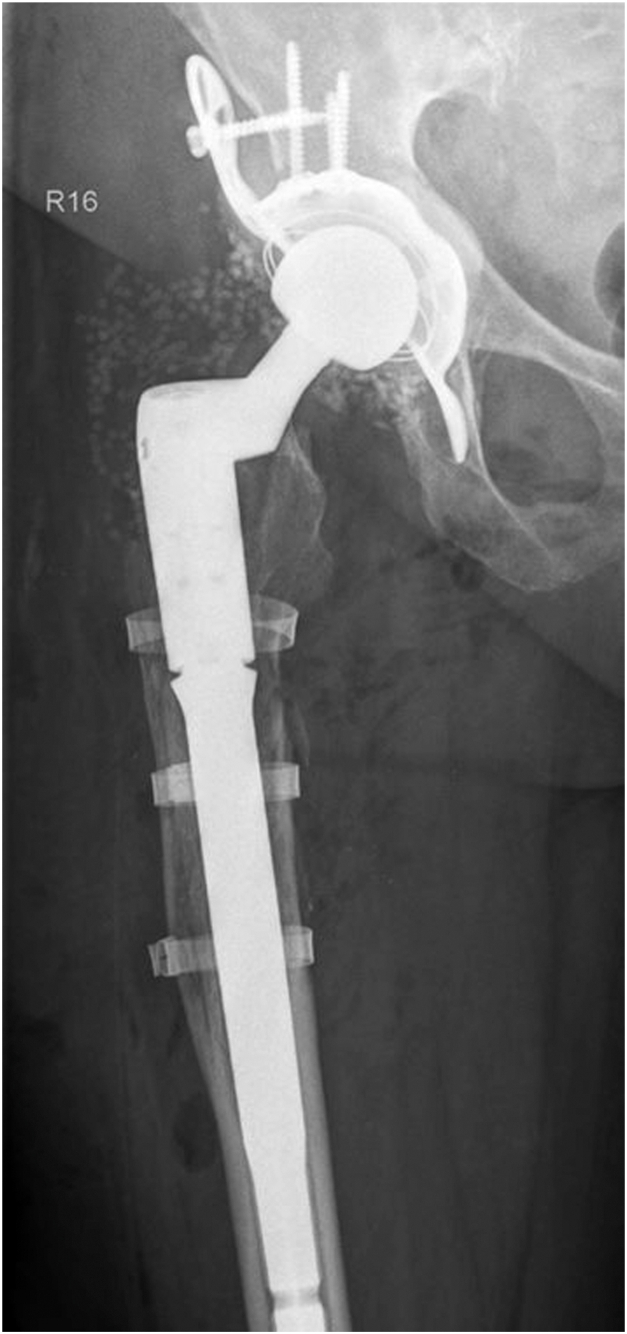
Postoperative anteroposterior radiograph of the right hip, postoperative imaging showcases the Burch-Schneider Ring, cemented polyethylene cup and modular cementless stem (Implants: Burch-Schneider Revision 56 mm, Fa. Zimmer, Warsaw, Indiana, US; Exeter X3 Rimfit 36/52 mm, Fa. Stryker, Kalamazoo, Michigan, US; Mutars modular stem 16/200 mm, middle piece 50 mm, head piece 135°/32 mm, Fa. Implantcast, Buxtehude, Germany; Biolox 36 mm ceramic head size XL, Fa. CeramTech, Plochingen, Germany).

The patient participated in early postsurgical physiotherapy sessions while fully weight-bearing in order to ensure a timely mobilization. This was successfully achieved on the first postoperative day. Postoperative X-rays confirmed a satisfactory position of the implants (Fig. 3).

The histopathological workup showed a periprosthetic membrane of the abrasion-induced type, severe metallosis, and also fractured trabecular material indicating an aseptic loosening of implant components [21].

Microbiological cultures obtained intraoperatively remained negative after 14 days of incubation.

### Postoperative complications

A wound revision was performed 9 days after surgery due to the formation of a seroma that extended epifascially and subfascially. Microbiological and histopathological specimens showed no signs of infection.

### Follow-up

Clinical and radiological follow-up visits were scheduled for 6 weeks and 6 months postoperatively

At 6 weeks postoperatively, the patient reported that she was experiencing no pain, but felt insecure during ambulation, using a walker most of the time.

On examination, she presented with a well-healed incision. The patient achieved 90 degrees of flexion and full extension of her right hip, 20 degrees of external and 10 degrees of internal rotation, respectively. No new neurological deficit was present; however, reduced quadriceps muscle strength persisted to the extent noted preoperatively. There was no tenderness upon palpation, no Trendelenburg sign. A slight deficit in coordination was present.

The radiological findings showed a regular position of the implants. No new fractures or fragment dislocation were observed.

At 6 months postoperatively, the patient reported no pain regarding her hip, however, she still felt insecure while walking despite extensive participation in physiotherapy and exercise. An insecure and antalgic gait persisted, which was attributed mainly to the symptomatic arthritis of the patient’s right knee as well as the femoral nerve lesion that occurred during primary hip arthroplasty 20 years ago. Her range of motion increased slightly to 110 degrees of flexion and 20 degrees for internal and external rotation. There were no further changes compared to the follow-up after 6 weeks.

### Outcomes

The preoperative values of the Forgotten Joint Score worsened marginally from 64.6 to 60.4 points. The Oxford Hip Score decreased from 37 to 34 out of 48 possible points at 6 months postoperatively. While the change in her symptoms did not exceed the threshold for the minimal clinically important difference described in the literature, the patient noticed that she was not as mobile as before [22,23]. The health-related quality of life measured using the EQ5D-5D-5L questionnaire was 0.857 and amounted to 0.818 at 6 months postoperative.

### Patient perspective

The patient reported that she still experienced stiffness and felt insecure in her gait postoperatively. However, she also acknowledged that the risk of developing even larger bony defects in a nonoperative approach would not have been acceptable for her.

## Discussion

While earlier reports described promising results [13,14], the majority of retrospective reviews have since shown unacceptably high rates of failure in sandwich type inserts, with a fracture rate of up to 18 percent [16,[24], [25], [26]].

Risk factors for ceramic liner fracture as featured in the literature include a malpositioning of the cup or stem, impingement of implant components, a high body mass index, and high patient activity [16,24,27]. Our patient showed none of the described risk factors.

Also, our patient did not experience any symptoms regarding her hip. This is a rare clinical presentation of this pathology, as patients with a ceramic liner fracture usually experience considerable pain [11]. In a literature review performed by Wu et al., the vast majority of patients with a liner fracture reported significant discomfort [28].

If undetected, ceramic fragments will cause accelerated wear, thereby leading to metallosis, osteolysis and, ultimately, loosening of implant components [7]. Cases of catastrophic osteolysis and protrusion of the femoral head through the acetabular shell and into an intrapelvine position have been reported independently by Winston and Bekler [29,30]. In the case presented by Winston et al., intervention had been delayed by approximately 10 years [29]. However, in the case by Bekler et al., fracture and intrapelvine protrusion of the components occurred early at 14 months postoperatively [30]. In our case, liner fracture had been present for at least 4 years.

Early diagnosis and intervention are therefore essential in cases of ceramic liner fracture. While notching of the femoral neck has been described before [31], we believe naming this phenomenon may further increase awareness for both ceramic liner fracture and notching due to implant impingement. We propose naming this phenomenon the Femoral Neck-Bite Sign as an indicator of a ceramic liner fracture or impingement of implant components.

It may showcase the abrasive effect of the ceramic fragments and can act as an additional radiographic finding, even if an actual fracture cannot be observed. In this case, the damaged implant had not been appreciated earlier.

Severe notching of the femoral neck can compromise implant stability and necessitate stem revision [18]. Additionally, if observed, a significant amount of intraarticular metallosis may be assumed, which may be associated with osteolysis and aseptic loosening of the implant will be more likely. This can be used in preoperative planning and intraoperative decision-making, thereby influencing the therapeutic course of action. It should be noted that some extent of notching of the femoral neck can also be present in cases of ceramic liner impingement and may not be confused with a liner fracture [31]. Kim et al. reported a high incidence of femoral notching in a cohort of 456 total hip arthroplasties performed with a thick-neck stem design, none of those 49 patients received revision surgery for isolated femoral notching [31].

There are several points of debate regarding the course of treatment after ceramic liner fracture:

Most authors recommend removal of acetabular liner and femoral head, thorough debridement and removal of fragments as well as the introduction of a ceramic-on-ceramic or ceramic-on-polyethylene bearing to minimize third-particle wear [15,27,32,33]. The use of metal bearing components is contraindicated, cases of severe, even fatal, metallosis have been reported in abundance [29,[33], [34], [35], [36], [37], [38]]. Rambani et al. performed a literature review of 199 publications regarding revision total hip arthroplasty for fractured ceramic bearings, recommending implant removal if extensive damage to the components or component malpositioning is observed intraoperatively [32].

In the present case, large osseous defects made a removal of all implant components necessary and required complex acetabular reconstruction with a cage. This in turn necessitated the use of a cross-linked polyethylene cup that was used in accordance with national and international guidelines [18,29,32,39].

During the follow-up visits, the patient noted that the postoperative outcome was slightly worse than her preoperative state. This raises the question, if a nonoperative regimen would not have been more appropriate in this specific case.

In our opinion, the risk of further wear and development of even larger osteolytic areas was high. A failure of the arthroplasty in the near future seemed inevitable and even greater osseous defects were expected. Therefore we assumed that a revision at a later time would have been associated with an increased intraoperative risk and a worse functional outcome for the patient. This is concurrent with the literature, with cases showing protruding femoral components in an intrapelvine position, concomitant fractures, and systemic toxic metallosis [29,30,34,36,40].

## Summary

- Ceramic liner fracture is a rare but clinically important pathology which may be indicated by the Femoral Neck-Bite Sign in anteroposterior or axial radiographs of the hip.
- Presence of the Femoral Neck-Bite Sign should warrant further investigation.
- If a fracture in a ceramic type of bearing is confirmed, a timely revision surgery is usually indicated.
- Whenever possible, implant retention and removal of interchangeable parts with introduction of a ceramic-on-ceramic or ceramic-on-cross-linked polyethylene bearing should be performed.
- Large bony defects may be present and may necessitate a total removal of components and use of modular implants.

## Conflicts of interest

R. Stauss received speakers bureau/paid presentations for Artiqo and received research support from Smith & Nephew as a Principal Investigator. P. Savov received speakers bureau/paid presentations for Smith & Nephew and Microport and is a paid consultant for Smith & Nephew and Microport. M. Ettinger received speakers bureau/paid presentations for Smith & Nephew and Microport; is a paid consultant for Smith & Nephew and Microport; and received research support from Smith & Nephew as a Principal Investigator. All other authors declare no potential conflicts of interest.

For full disclosure statements refer to https://doi.org/10.1016/j.artd.2025.101740.

## Informed patient consent

The author(s) confirm that written informed consent has been obtained from the involved patient(s) or if appropriate from the parent, guardian, power of attorney of the involved patient(s); and, they have given approval for this information to be published in this case report (series).

## CRediT authorship contribution statement

**Hendrik Pott:** Writing – review & editing, Writing – original draft, Visualization, Software, Resources, Project administration, Methodology, Investigation, Data curation, Conceptualization. **Ricarda Stauss:** Writing – review & editing, Writing – original draft, Supervision, Project administration, Investigation. **Peter Savov:** Writing – review & editing, Writing – original draft, Supervision, Project administration, Investigation. **Max Ettinger:** Writing – review & editing, Writing – original draft, Supervision, Investigation, Data curation. **Ralf Dieckmann:** Writing – review & editing, Writing – original draft, Validation, Supervision, Software, Resources, Project administration, Methodology, Investigation, Formal analysis, Data curation, Conceptualization.

## Appendix A Supplementary Data

Conflict of Interest Statement for Pott

Conflict of Interest Statement for Ettinger

Conflict of Interest Statement for Savov

Conflict of Interest Statement for Dieckmann

Conflict of Interest Statement for Stauss
