# Supplementary material for: The Femoral Neck-Bite Sign: A Radiographic Indicator of Catastrophic Sandwich Liner Failure in Total Hip Arthroplasty
Source: Arthroplast Today. 2025 Jun 23;34:101740. doi: 10.1016/j.artd.2025.101740 (PMC12240126; doi:10.1016/j.artd.2025.101740)
Supplement: Conflict of Interest Statement for Savov [file mmc3.pdf]

CONFLICT OF INTEREST STATEMENT

American Association of Hip and Knee Surgeons

(Adopted from the American Academy of Orthopaedic Surgeons disclosure statement)

The following form**must be filled out completely and submitted by each author (example, 6 authors, 6 forms). All items require a response. If there is no relevant disclosure for a given item, enter "*None.*"**

Manuscript Title

The Femoral Neck-Bite Sign: A radiological finding indicating catastrophic failure of a sandwich liner in total hip arthroplasty – A case report

1. Royalties from a company or supplier (The following conflicts were disclosed)

   ∅

2. Speakers bureau/paid presentations for a company or supplier (The following conflicts were disclosed)

   Smith & Nephew, Microport

3A. Paid employee for a company or supplier (The following conflicts were disclosed)

   ∅

3B. Paid consultant for a company or supplier (The following conflicts were disclosed)

   Smith & Nephew, Microport

3C. Unpaid consultants for a company or supplier (The following conflicts were disclosed)

   ∅

4. Stock or stock options in a company or supplier (The following conflicts were disclosed)

   ∅

5. Research support from a company or supplier as a Principal Investigator (The following conflicts were disclosed)

   ∅

6. Other financial or material support from a company or supplier (The following conflicts were disclosed)

   ∅

7. Royalties, financial or material support from publishers (The following conflicts were disclosed)

   ∅

8. Medical/Orthopaedic publications editorial/governing board (The following conflicts were disclosed)

   ∅

9. Board member/committee appointments for a society (The following conflicts were disclosed)

   ∅

Each author must sign AND print or type his/her name, date and submit a separate form

In addition, one BLINDED Conflict of Interest form (no author names used) should be submitted per manuscript with all author disclosures.

Priv.-Doz. Dr. med. Peter Savov		25.2.25
Author Name (Print or Type)	Author Signature	Date
